# DNA Demethylation of Promoter Region Facilitates Atoh-1-Induced Interleukin-19 Expression Activation in Bone Marrow Monocytes of Old Mice

**DOI:** 10.14336/AD.2024.0108

**Published:** 2024-01-08

**Authors:** Yanan Chen, Longshuai Lin, Shengzhe Ruan, Jianxin Ye, Zhicheng Dai, Guangzhen Mao, Yongming Xi, Changping Wang, Qinghua Zhao

**Affiliations:** ^1^Department of Orthopedics, Shanghai General Hospital, Shanghai Jiao Tong University School of Medicine, Shanghai, China.; ^2^Precision Research Center for Refractory Diseases, Institute for Clinical Research, Shanghai General Hospital, Shanghai Jiao Tong University School of Medicine, Shanghai, China.; ^3^School of Medical Instrument and Food Engineering, University of Shanghai for Science and Technology, Shanghai, China.; ^4^Department of Spinal Surgery, The Affiliated Hospital of Qingdao University, Qingdao, Shandong, China

**Keywords:** Age, BMMs, Interleukin-19, DNA demethylation, Atoh-1

## Abstract

With increasing age, there is a notable increase in the differentiation of bone marrow-derived mononuclear cells (BMMs) into osteoclasts, accompanied by a concurrent rise in both osteoclast quantity and activity. This escalation in osteoclastic activity accelerates bone resorption, which in turn contributes to age-related bone loss and metabolic bone disorders, notably osteoporosis. Our study confirms that elevated IL-19 expression promotes aging-induced bone loss in aged mice and sheds light on the regulatory mechanisms upstream of IL-19 expression and secretion. Primarily, it is the methylation status of the IL-19 gene’s promoter region that impacts Atonal BHLH Transcription Factor 1 (Atoh1)'s ability to bind to the promoter. We found that this specific mechanism involves reduced expression and binding affinity of Dnmt1 to the IL-19 promoter region. The findings of our study suggest that targeting IL-19 could be a potential strategy for managing bone loss-related conditions and enhance the current understanding of how DNA methylation levels contribute to age-related bone loss.

## INTRODUCTION

Monocyte/macrophage lineage cells are highly plastic and can differentiate into a variety of cells in response to different environmental stimuli [1]. Osteoclasts, specialized multinucleated cells that originate from this lineage, play a pivotal role in bone resorption, facilitating both the development and ongoing remodeling of bone [1, 2]. Intercellular fusion and multinucleation of BMMs are essential processes for osteoclast maturation and bone resorption capacity [3-5]. This fusion process requires the presence of macrophage colony-stimulating factor (M-CSF) and receptor activator of nuclear factor (NF)-κB ligand (RANKL), and is regulated by many signaling factors such as cytokines, chemokines, and transcription factors [1, 6, 7]. With increasing age, there is a marked increase in the differentiation of BMMs into osteoclasts. This process leads to a rise in both the number and activity of osteoclasts, resulting in enhanced bone resorption. Subsequently, this increase in bone resorption contributes to age-related bone loss and disorders of bone metabolism, with osteoporosis being particularly prevalent [8-10]. In individuals with severe osteoporosis, the incidence of fragility fractures is high, representing a potentially life-threatening complication for the elderly [11]. Therefore, a deeper understanding of the molecular underpinnings of age-related bone loss is essential to advance therapeutic strategies.

IL-19, a member of the IL-20 subfamily within the IL-10 cytokine family, along with IL-20 and IL-24, orchestrates cellular responses by inducing hetero-dimerization of the IL-20R1/IL-20R2 (type I) receptors [12, 13]. This cytokine is predominantly produced by myeloid cells, T-cells and epithelial cells in reaction to either physiological or pathological stimuli, predominantly affecting epithelial cells and monocytes [14]. Furthermore, research has revealed that osteocytes are a significant source of IL-19 as well [15]. Similar to its relatives IL-20 and IL-24, IL-19 has both anti-inflammatory and pro-inflammatory effects and is implicated in the pathology of certain inflammatory bone conditions [13, 16, 17]. It has been demonstrated that IL-20 and IL-24 enhance osteoblast activity, and IL-20 regulates osteoclast differentiation through multiple pathways [17].In addition, the expression and secretion of interleukin family members are modulated by histone epigenetics and DNA modifications, particularly DNA methylation patterns [18, 19]. Evidence has shown that DNA methylation of CpG regulates IL-19 in CD4 ^+^ T cells and macrophages [20]. Nonetheless, the mechanisms controlling IL-19 expression and secretion, as well as its function in bone metabolism, are complex and warrant more thorough investigation.

Epigenetic regulation extends beyond the secretion of interleukins, with an expanding volume of research indicating its involvement in the control of bone metabolism and its impact on the differentiation of osteoblasts and osteoclasts [21-25]. Epigenetics, commonly understood as heritable alterations in phenotype without changes to the DNA sequence, encompasses mechanisms such as DNA methylation, histone modifications, and noncoding RNAs (ncRNAs) [26]. These mechanisms play a pivotal role in the cellular differentiation program and exhibit dynamic changes with aging [21, 26]. Notably, DNA methylation typically occurs at the 5-position of cytosine rings within CpG dinucleotides, leading to the formation of 5-methylcytosine (5mC), and is a crucial epigenetic modification [19, 20]. Molecular and genetic studies in mammals have shown that DNA methylation is associated with gene silencing and is frequently linked to transcriptional repression of transposon factors [27, 28]. The enzymatic process of DNA methylation is facilitated by DNA methyltransferases (Dnmts), which play a central role in epigenetic gene regulation [29]. The human genome encodes five Dnmts: Dnmt1, Dnmt2, Dnmt3a, Dnmt3b and Dnmt3l, of which Dnmt2 and Dnmt3l lack catalytic activity [30]. The Dnmt3 family members are critical for setting up new methylation patterns and mediating de novo methylation, whereas Dnmt1 maintains these methylation patterns across cell division [31].

In our study, we have pinpointed the role of IL-19 in age-related bone loss and its methylation-dependent regulatory mechanisms. We demonstrated that the expression and secretion levels of IL-19 in mouse BMMs increased with age, and that BMMs-specific knockdown of IL-19 alleviated bone loss and osteoporosis in old mice. Then, we elucidated that the increased IL-19 expression and secretion in old mice was mainly caused by the hypomethylation level of the promoter region of the IL-19 gene, and the specific mechanism was due to the decreased expression level and binding level to the promoter region of IL-19 by Dnmt1. The role of Dnmt1 was obtained by 5'-aza inhibition and overexpression of Dnmt1 in the transfection experiment validated. Moreover, we uncovered that demethylation of DNA in the IL-19 promoter activated IL-19 expression by promoting Atonal BHLH Transcription Factor 1(Atoh1) binding at the IL-19 promoter.

## MATERIALS AND METHODS

### Animal studies

All mice were maintained under SPF conditions in the animal facility of Shanghai General Hospital. All experiments were performed with the protocol approved by the Animal Care and Use Committee of Shanghai General Hospital. C57BL/6 mice were purchased from SIPPR-BK Laboratory Animal Co. Ltd (Shanghai, China). IL-19^flox/flox^ mice were generated by inserting loxP sequence into upstream of exon 1 and downstream of exon 3 of IL-19 gene. IL-19^flox/flox^ mice were bred with transgenic LysM-Cre mice (Stock No: # 004781, Jackson Laboratory, Bar Harbor, USA) to yield IL-19^LysM-Cre^ mice, in which IL-19 functions in BMMs were canceled due to deletion of exon 1 and 3 of IL-19 gene in BMMs.

Six male animals were included per group. Animals were euthanized using isoflurane inhalation anesthesia followed by cervical dislocation. Lumbar vertebrae #1 and left femur were prepared for micro computed tomography (micro-CT) scan. The right femur and bilateral tibias were prepared for bone marrow aspirates. Peripheral blood serum was prepared for the measurement of IL-19 levels.

### BMMs isolation, culture, and treatment

Primary BMMs were isolated from mice as previously done [10]. Animals were euthanized using isoflurane inhalation anesthesia followed by cervical dislocation. Then, the bone marrow cells were flushed out from femur and tibia bones with ɑ-Minimum Essential Medium (MEM) (HyClone; Danaher Corporation, USA). Cell suspensions were filtered through a 100 µm cell strainer (Falcon; Corning, USA) and cultured in ɑ-MEM supplemented with 10% fetal bovine serum (FBS) (MilliporeSigma, USA), 1% penicillin/streptomycin (Invitrogen; Thermo Fisher Scientific, USA), and 1% GlutaMAX Supplement (Thermo Fisher Scientific) for

## 24 hours. The supernatant was collected, and cell precipitation was obtained by centrifugation, then BMMs were cultured in ɑ-MEM medium supplemented with 10% FBS, 1% GlutaMAX Supplement, and 20 ng/ml M-CSF (PeproTech, Thermo Fisher Scientific) in a humidified atmosphere at 37°C and 5% CO2. BMMs were harvested on Day 2 after M-CSF stimuli.

To inhibit the activity of DNA methyltransferase, we employed 10 μM 5’-aza (#50646, Cell Signaling Technology, Danvers, MA, USA) to treat BMMs.

### Quantitative reverse-transcriptase PCR (RT-qPCR)

Total RNA was extracted using Trizol Reagent (Invitrogen) according to the manufacture’s protocol and reverse-transcribed into cDNA using a First Strand cDNA Synthesis Kit (Invitrogen). The details of the procedure were as follows: sequentially added 4 µL of 5X Reaction Mix, 2 µL of Maxima Mixed Enzyme, 1ug of template RNA, and supplemented with nucleic acid-containing water to 20ul of the system to a sterile, RNAase-free EP tube on ice. Then, gently mixed and centrifuged. Incubated at 25°C for 10 minutes, then at 50°C for 15 minutes, and finally heated at 85°C for 5 minutes to terminate the reaction and reverse-transcribed into cDNA.

The thermocycling conditions were as follows: initial denaturation at 95°C for 30 sec, followed by 40 cycles of 95°C for 15 sec, 60°C for 60 sec. The melting curve was generated to determine normality. SYBR Green Master (Takara) was used for the quantitative analysis. β-actin was used as the internal reference gene. The 2-ΔΔCt method was used to analyze the results. The primer sequences of the genes are presented in Table S1.

### Western blot

Cells were lysed on ice for 30 min in a lysis buffer (50 mM Tris-HCl, 150 mM NaCl, 1% Nonidet P-40, and 0.1% SDS supplemented with protease inhibitors). The protein samples were loaded into the SDS polyacrylamide gel electrophoresis (SDS-PAGE) gels and then transferred onto nitrocellulose membranes (Axygen, USA). Membranes were blocked with 5% skimmed milk at room temperature for 1 h and incubated with primary antibodies at 4 °C overnight, following by incubation with the secondary antibodies at room temperature for 1 h. Finally, the membranes were visualized with an Enhanced Chemiluminescence (ECL) Detection Kit (MilliporeSigma, USA) and by using an enhanced chemiluminescence system (GE Healthcare, Piscataway, NJ, USA). The western blot used was 12% SDS-PAGE with a loading volume of 20 μg per well, and the antibody stock numbers were as follows: Atoh1 rabbit mAb (1/1000; PA5-76016, Thermo Fisher), His tag rabbit mAb (1:1000; #12698, Cell signaling technology), HA tag rabbit mAb (1:1000; #3724, Cell signaling technology) and β-actin rabbit mAb (1:1000; #4970, Cell signaling technology). The secondary antibody used was the anti-rabbit horseradish peroxidase (HRP)-conjugated antibody (1/5000; #S0001, affinity biosciences).

### ELISA

The blood collected from mice was centrifuged for 30 min at 2,000g and the plasma was collected and stored at -80 °C for subsequent assays. IL-19 levels in the peripheral blood serum, bone marrow plasma or the supernatants of in vitro BMMs culture of mice were measured with ELISA kits (NBP3-06800, Novus Biologicals) according to the manufacturer’s instructions, and the steps were as follows. Add the capture antibody solution to a PVC transparent microtiter plate, incubate the plate at 4°C overnight and blot out the liquid in the wells. Wash three times with wash buffer. reagent dilutions were added, the plate was blocked and incubated for 60 minutes at room temperature before ejecting the solution and washing three times with wash buffer. Next, diluted samples and standards are added, the plate is capped and incubated for 2 hours at room temperature before the solution is aspirated and washed three times with Wash Buffer. Then add the detection antibody solution, incubate at room temperature for 2 hours, then eject the solution and wash three times with wash buffer. A substrate solution is added and incubated for 20-30 minutes at room temperature, then absorbance is measured immediately using a spectrophotometer.

### Micro-CT

Bone densities of femur and L1 vertebrae were measured by micro-CT (μCT-80, Scanco Medical AG, Bassersdorf, Switzerland). Standard nomenclature and guidelines for bone microstructure were employed 1. The bones were scanned at an energy level of 55 kVp, intensity of 145μA, and a fixed threshold of 220. Trabecular regions of the whole L1 vertebrae were analyzed. The trabecular region of femur was analyzed at 0.35mm from the growth plate of distal femur. Three-dimensional images were reconstructed. The main parameters of trabecular bone are BV/TV (bone volume/total volume), Tb.N (trabecular bone number) and Tb.Sp (trabecular bone space).

### Bone histomorphometry

Bone histomorphometric analysis to quantify osteoclasts was performed in mouse femurs embedded in paraffin that were stained for Trap. Osteoclasts were identified as multinucleated Trap-positive cells adjacent to bone surface. All analysis was confined to the secondary spongiosa and restricted to an area between 500 and 2000 μm proximal to the growth plate-metaphyseal junction of the distal femur. The number of osteoclasts around the bone surface was calculated. Five sections of each staining were analyzed per animal. Images were analyzed using ImageJ in a blinded manner.

### Bisulphite sequencing PCR

Bisulfite-treated genomic DNA of mouse BMMs were amplified by PCR with BSP forward primer 5’-GTGTGAAGTTGTGGTTTTTA-3’ and reverse primer 5’-TAATTCTCTAACAAAACAAA-3’, which amplified the promoter region (-441 bp to -56 bp) of mouse IL-19 gene. The amplification procedure consisted of 5 min. of denaturation at 94°C followed by 25 to 30 cycles at 94°C (1 min.), 55-57°C (30 sec.), and 72°C (30 sec.), ending with 10 min. of extension at 72°C. The amplified PCR products were gel-purified with a PCR Product Purification Kit (Sangon Biotech, China) and cloned into pMD-19T vector system (Promega, USA). Ten colonies from each PCR reaction were randomly chosen for sequencing and the percentages of methylated cytosines over total cytosines within the cloned fragment were calculated.

### Chromatin immunoprecipitation (ChIP)

Cells were fixed with 1% formaldehyde for 10 min at 37 °C and nuclei were prepared. The crude nuclei were subjected to sonication to produce chromatin fragments with approximately 400 bp of length. 2-5 μg primary antibodies including anti-Dnmt1 (MA5-35340, Thermo Fisher), anti-Dnmt3a (MA5-16171, Thermo Fisher), anti-Dnmt3b (NB300-516, Novus Biologicals), anti-Dnmt3l (orb340835, Biorbyt) and isotype IgG as negative control (#61656, Cell signaling technology) and the samples were incubated overnight at 4 °C with gentle shaking. Primer sequences for ChIP-qPCR were forward primer 5’-GGACCTGGTCAGAAATGGGT-3’ and reverse primer 5’-AGCAGGAGGCAGCTCTTGTC-3’.

### Luciferase reporter assay

A 472 bp (-470 bp/+2 bp) fragment of mouse IL-19 promoter was amplified from BMMs. This fragment was cloned into the plasmid pGL3-Basic (Promega, CharbonniËres, France). Cells were seeded into 24-well plates. All plasmids were isolated by QIAGEN plasmid purification kit (QIAGEN). Transient transfection by lipofectamine3000 (Invitrogen) was performed and phRL-SV40 vector (Promega) was used as transfection efficiency control. Forty-eight hours after transfection, both firefly and renilla luciferase activities were measured using Dual-luciferase reporter assay system (Promega).

### In vitro methylation assay

A 472 bp (-470 bp/+2 bp) fragment of mouse IL-19 promoter was amplified and then purified by gel electrophoresis and methylated at all CpG sites with M.SssI (2U/g of DNA, 6hrs at 37°C), or mock methylated. M.SssI was then inactivated at 65°C for 15mins. Treated DNA fragments and vectors were ligated and purified by phenol-chloroform extraction and ethanol precipitation. The plasmid concentrations were determined by measuring the A260.

**Figure 1.**
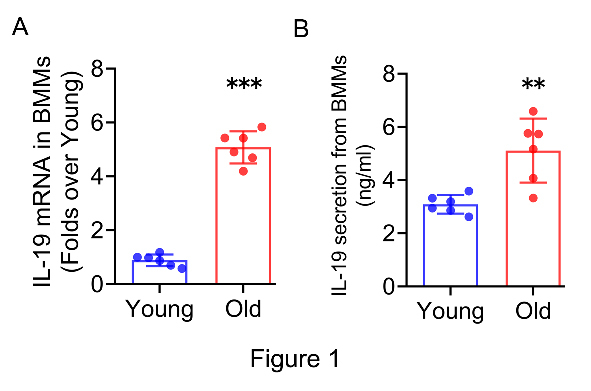
**IL-19 expression up-regulation in BMMs from old mice**. (**A**) IL-19 mRNA expression in BMMs from young and old mice determined by RT-qPCR. (**B**) IL-19 secretion levels in the supernatant of BMMs from young and old mice determined by ELISA. (A, B, n=6 per group; one technical replicate of six biological replicates for each group). β-actin was used as internal control for RT-qPCR. Data are representative of three independent experiments. Data were shown as the means ± s.d. P-values were determined by unpaired two-tailed Student’s t-test (A, B). **: p<0.01, ***: p<0.001, Old vs. Young.

### Statistical analysis

All data are expressed as mean ± s.d. All data were tested for normality using the Shapiro-Wilk test. For data that conformed to normal distribution, data between two groups were compared using unpaired Student's t-test, and data from three and more groups were analyzed by one-way analysis of variance (ANOVA) for multiple comparisons. ANOVA was analyzed using the Turkish test for statistically significant differences. For data that did not conform to normal distribution, we used nonparametric tests and Mann-Whitney *U*-test for data between two groups. Normality tests were performed using SPSS 16.0 software, and statistical analyses and graphs were generated using GraphPad Prism 7 software. P-values (or adjusted P-values) <0.05 were considered statistically significant.

Biological replicates refer to the experiments conducted by the same treatment between samples of different biological individuals or different biological groups, whereas technical replicates refer to multiple identical experiments conducted on the same sample. All specific statistical details and the number of biological or technical replicates can be found in the figure legends.

**Figure 2.**
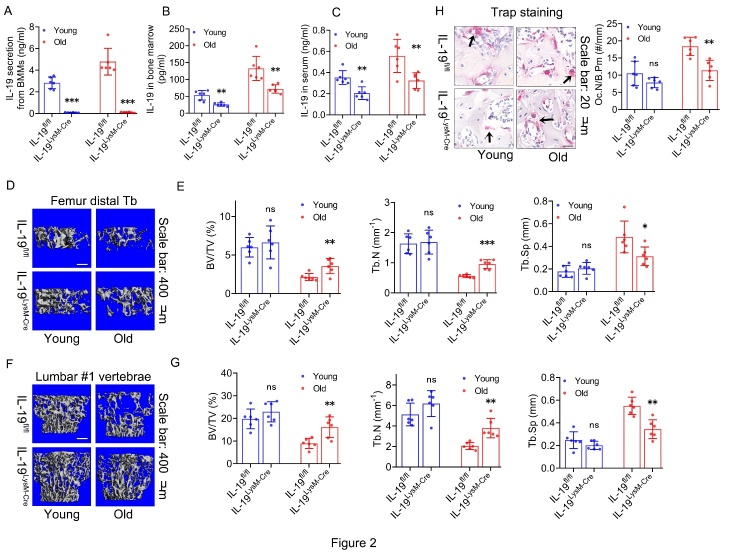
**Alleviation of trabecular bone loss of old mice in response to in vivo deletion of IL-19 from BMMs**. (**A**) IL-19 secretion levels in the supernatant of BMMs from young and old mice with and without in vivo IL-19 deletion from BMMs determined by ELISA. (**B**) IL-19 levels in the bone marrow of young and old mice with and without in vivo IL-19 deletion from BMMs determined by ELISA. (**C**) IL-19 levels in the peripheral blood serum of young and old mice with and without in vivo IL-19 deletion from BMMs determined by ELISA. (**D**) Representative Micro-CT 3D reconstruction images of trabecular bone in the distal femur from young and old mice with and without in vivo IL-19 deletion from BMMs. (**E**) Quantification of trabecular bone volume fraction (BV/TV), trabecular bone number (Tb.N), and trabecular bone separation (Tb.Sp) of trabecular bone in the distal femur from young and old mice with and without in vivo IL-19 deletion from BMMs determined by Micro-CT. (**F**) Representative Micro-CT 3D reconstruction images of trabecular bone in lumbar #1 (L1) vertebrae from young and old mice with and without in vivo IL-19 deletion from BMMs. (**G**) Quantification of BV/TV, Tb.N, and Tb.Sp of trabecular bone in L1 vertebrae from young and old mice with and without in vivo IL-19 deletion from BMMs determined by Micro-C. (**H**) Representative Trap staining and quantification of osteoclast number (Oc.N) of in the distal femur from young and old mice with and without in vivo IL-19 deletion from BMMs. (A-H, n=6 per group; one technical replicate of six biological replicates for each group). The arrows point at the positive staining. Data are representative of three independent experiments. Data were shown as the means ± s.d. P-values were determined by unpaired two-tailed Student’s t-test (B, E, G, H), and unpaired two-tailed Mann-Whitney U-test (A, C). *: p<0.05, **: p<0.01, ***: p<0.001, ns: no significance, IL-19^LysM-Cre^ vs. IL-19^fl/fl^.

**Figure 3.**
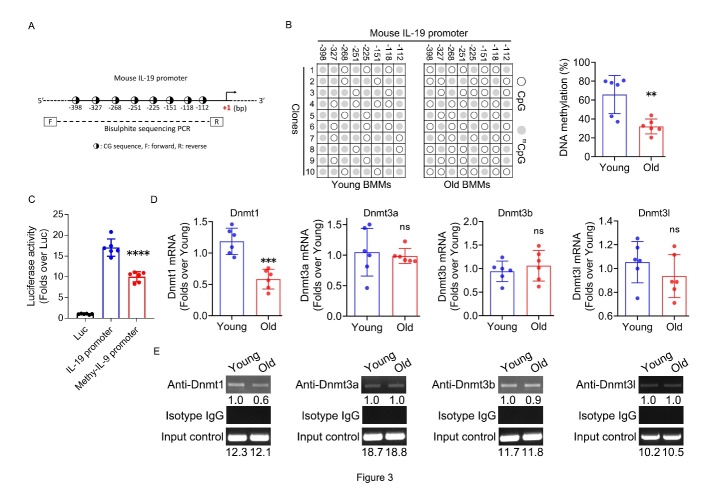
**DNA demethylation of IL-19 promoter and Dnmt1 down-regulation in BMMs from old mice**. (**A**) Diagram of mouse IL-19 promoter. ◑: CG sequence. (**B**) DNA methylation percentage of 8 CG sites within mouse IL-19 proximal promoter in BMMs from young and old mice determined by bisulphite sequencing PCR. (**C**) Luciferase activity of un-methylated and methylated mouse IL-19 promoter-driven luciferase reporter gene vector in BMMs. (**D**) Dnmt1, Dnmt3a, Dnmt3b, and Dnmt3l mRNA expression in BMMs from young and old mice determined by RT-qPCR. (**E**) Dnmt1, Dnmt3a, Dnmt3b, and Dnmt3l binding on mouse IL-19 promoter in BMMs from young and old mice determined by ChIP. β-actin was used as internal control for RT-qPCR. (A-D, n=6 per group; one technical replicate of six biological replicates for each group; E, representative results from two independent biological experiments). Data are representative of three independent experiments. Data were shown as the means ± s.d. P-values were determined by unpaired two-tailed Mann-Whitney U-test (B and Dnmt3a group of D), unpaired two-tailed Student’s t-test (other groups of D), and one-way analysis of variance (ANOVA) followed by Tukey’s test (**C**). **: p<0.01, ***: p<0.001, ****: adjusted p<0.0001, ns: no significance, Old vs. Young, Methyl-IL-19 promoter vs. IL-19 promoter.

## RESULTS

### IL-19 expression up-regulation in BMMs from old mice.

To ascertain the differential expression of IL-19 in BMMs between young (5-month-old) and old (16-month-old) mice, we harvested BMMs from both age groups for in vitro cultivation and measured IL-19 mRNA expression and secretion levels. RT-qPCR analysis revealed a substantial increase in IL-19 mRNA levels in BMMs from old mice in comparison to those from young mice (Fig. 1A). In alignment with the mRNA data, ELISA results indicated that IL-19 secretion was elevated in older mice relative to their younger counterparts (Fig. 1B). Collectively, these findings corroborate the upregulation of IL-19 expression in BMMs derived from old mice.

### Alleviation of trabecular bone loss of old mice in response to in vivo deletion of IL-19 from BMMs.

Considering the potential role of IL-19 in age-related bone loss, we engineered BMMs-conditioned knockout IL-19 (IL-19^LysM-Cre^) mice and used IL-19^fl/fl^ mice as controls. We discovered that BMMs from both young and old IL-19^LysM-Cre^ mice ceased to secrete IL-19 when cultured *in vitro* (Fig. 2A). This finding was supported by subsequent observations that the levels of IL-19 in bone marrow suspensions and peripheral blood were markedly reduced in IL-19^LysM-Cre^ mice compared with IL-19^fl/fl^ mice (Fig. 2B and 2C). Micro-CT analysis of distal femoral trabecular bone (Fig. 2D) and L1 vertebral trabecular bone (Fig. 2F) showed a significant increase in bone mass in old IL-19^LysM-Cre^ mice compared with IL-19^fl/fl^ mice, characterized with increased BV/TV, Tb.N, and decreased Tb.Sp (Fig. 2E and 2G), whereas no significant changes were observed in young IL-19^LysM-Cre^ mice. Furthermore, lower number of TRAP-positive osteoclasts than IL-19^fl/fl^ mice was shown by TRAP staining on paraffin-embedded bone sections was observed on the surface of trabecular bone in old IL-19^LysM-Cre^ mice, with no change in young mice (Fig. 2H). Collectively, these findings suggest that the deletion of IL-19 in BMMs mitigates trabecular bone loss in aged mice. However, the mechanisms underlying this protective effect are not solely due to the suppression of osteoclast differentiation, and the potential influences on osteoblasts and on the broader bone microenvironment also require further investigation.

### DNA demethylation of IL-19 promoter and Dnmt1 down-regulation in BMMs from old mice.

Abnormal DNA methylation can impact gene expression, and in light of potential DNA methylation effects on IL-19 levels in old mice, we examined the CG sequences within the proximal promoter of the mouse IL-19 gene for potential DNA methylation sites (Fig. 3A). To assess the differences in the DNA methylation levels within the proximal promoter region of the IL-19 gene, we isolated nuclear genomic DNA from BMMs of both young and old mice. Bisulfite sequencing PCR analysis revealed that the proximal promoter region of the IL-19 gene was hypomethylated in BMMs from old mice compared to those from young mice (Fig. 3B). The IL-19 gene promoter region of the nuclear genomic DNA of BMMs was amplified by PCR at -470 bp/+2 bp, and the region was cloned into the pGL-3 Basic vector to construct an IL-19 promoter-driven luciferase reporter gene vector. The IL-19 promoter sequence was methylated in vitro using M.SssI enzyme, followed by transient transfection of mouse BMMs. The resultant luciferase assay indicated that methylation of the IL-19 gene promoter markedly reduced its transcriptional activity in BMMs (Fig. 3C).

**Figure 4.**
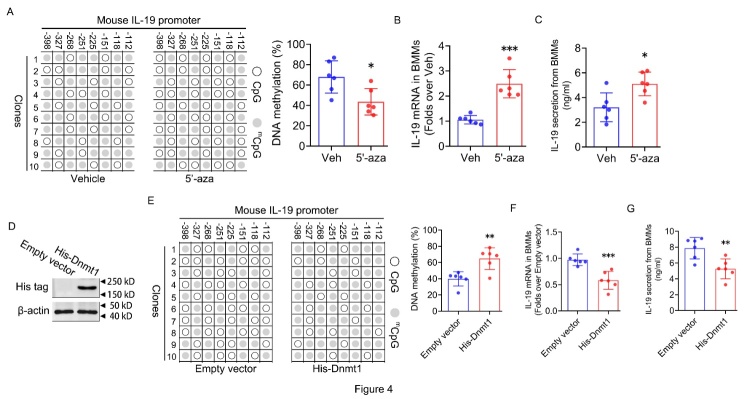
**Involvement of DNA demethylation of promoter region in IL-19 expression regulation in BMMs**. (**A**) DNA methylation percentage of 8 CG sites within mouse IL-19 proximal promoter in young BMMs with and without 5’-aza (10μM) treatment determined by bisulphite sequencing PCR. (**B**) IL-19 mRNA expression in young BMMs with and without 5’-aza (10μM) treatment determined by RT-qPCR. (**C**) IL-19 secretion levels in the supernatant of young BMMs with and without 5’-aza (10μM) treatment determined by ELISA. (**D**) Dnmt1 protein expression in old BMMs in response to exogenous His-tagged Dnmt1 over-expression determined by western blot. (**E**) DNA methylation percentage of 8 CG sites within mouse IL-18 proximal promoter in old BMMs in response to exogenous His-tagged Dnmt1 over-expression determined by bisulphite sequencing PCR. (**F**) IL-19 mRNA expression in old BMMs in response to exogenous His-tagged Dnmt1 over-expression determined by RT-qPCR. (**G**) IL-19 secretion levels in the supernatant of old BMMs in response to exogenous His-tagged Dnmt1 over-expression determined by ELISA. (A-C, G, n=6 per group; one technical replicate of six biological replicates for each group; D, representative results from two independent biological experiments). β-actin was used as internal control for RT-qPCR and western blot. Data are representative of three independent experiments. Data were shown as the means ± s.d. P-values were determined by unpaired two-tailed Mann-Whitney U-test (**E**) and unpaired two-tailed Student’s t-test (others)*: p<0.05, **: p<0.01, ***: p<0.001, 5’-aza vs. Vehicle, His-Dnmt1 vs. Empty vector.

Given that DNA methylation is dependent on the activity of DNA methyltransferases (DNMTs), we evaluated the mRNA expression levels of several major Dnmts in old and young mice. The mRNA expression level of Dnmt1 was significantly downregulated in BMMs of old mice compared to BMMs of young mice, whereas Dnmt3a, 3b and 3l were unchanged (Fig. 3D). To elucidate the correlation between Dnmts and IL-19 expression, we examined the binding levels of Dnmts to the IL-19 gene promoter in mouse BMMs. The binding affinity of Dnmt1 to the IL-19 gene promoter was substantially decreased in BMMs of old mice compared to BMMs of young mice, whereas the binding of Dnmt3a, 3b, and 3l remained constant (Fig. 3E). These results indicate that involvement of DNA demethylation of promoter region is involved in the up-regulation of IL-19 expression in BMMs in old mice, in which DNMT1 may play a significant role.

**Figure 5.**
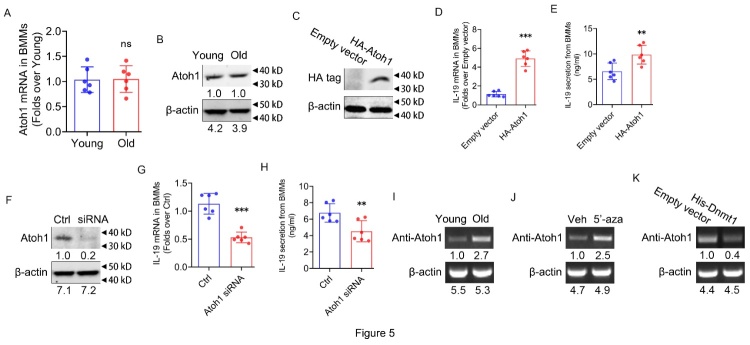
**Atoh-1 binding on IL-19 promoter to activate IL-19 expression due to DNA demethylation of IL-19 promoter**. (**A**) Atoh-1 mRNA expression in BMMs from young and old mice determined by RT-qPCR; n=6 per group. (**B**) Atoh-1 protein expression in BMMs from young and old mice determined by western blot. (**C**) Exogenous HA-tagged Atoh-1 protein expression in mouse BMMs in response to Atoh-1 over-expression determined by western blot. (**D**) IL-19 mRNA expression in mouse BMMs in response to exogenous Atoh-1 over-expression determined by RT-qPCR. (**E**) IL-19 secretion levels in the supernatant of BMMs in response to exogenous Atoh-1 over-expression determined by ELISA. (**F**) Atoh-1 protein expression in mouse BMMs in response to siRNA-mediated Atoh-1 knockdown determined by western blot. (**G**) IL-19 mRNA expression in mouse BMMs in response to siRNA-mediated Atoh-1 knockdown determined by RT-qPCR. (**H**) IL-19 secretion levels in the supernatant of BMMs in response to siRNA-mediated Atoh-1 knockdown determined by ELISA. (**I**) Atoh-1 binding on mouse IL-19 promoter in BMMs from young and old mice determined by ChIP. (**J**) Atoh-1 binding on mouse IL-19 promoter in BMMs with or without 5’-aza (10 μM) determined by ChIP. (**K**) Atoh-1 binding on mouse IL-19 promoter in BMMs in response to exogenous Dnmt1 over-expression determined by ChIP. (A, D, E, G, H, n=6 per group; one technical replicate of six biological replicates for each group; B, C, F, I-K, representative results from two independent biological experiments). β-actin was used as internal control for RT-qPCR and western blot. Data are representative of three independent experiments. Data were shown as the means ± s.d. P-values were determined by unpaired two-tailed Student’s t-test (A, D, E, G, H). **: p<0.01, ***: p<0.001, ns: no significance, Old vs. Young, HA-Atoh-1 vs. Empty vector, Atoh-1 siRNA vs. Ctrl.

### Involvement of DNA demethylation of promoter region in IL-19 expression regulation in BMMs.

Considering the involvement of DNMTs in the regulation of IL-19 expression associated with senescence, we investigated the impact of 5’-aza, a DNMT inhibitor known to impede enzymatic function by targeting DNMT1, on the expression and secretion of IL-19 in BMMs[32]. After a 48-hour exposure to 5’-aza, we observed a reduction in DNA methylation at the proximal promoter region of the IL-19 gene in treated mouse BMMs, with vehicle-treated mice serving as controls (Fig. 4A). In alignment with the methylation results, treatment of mouse BMMs with 5’-aza led to an upregulation of IL-19 mRNA expression (Fig. 4B) as well as an increase in its secretion (Fig. 4C). To further explore the effects of Dnmt1 on the DNA methylation level and expression level of IL-19, we constructed Dnmt1 overexpression plasmid vectors with His tags and transfected them into mouse BMMs. The successful introduction of exogenous His-Dnmt1 was confirmed via western blot (Fig. 4D). As expected, the overexpression of exogenous His-Dnmt1 protein in mouse BMMs increased the DNA methylation level of the proximal promoter region of the IL-19 gene and decreased the mRNA expression level and secretion level of IL-19 (Fig. 4E-G). Collectively, these findings suggest that the downregulation of Dnmt1 plays a crucial role in the demethylation of the IL-19 promoter region, thereby facilitating the upregulation of IL-19 expression in BMMs from old mice.

### Atoh-1 binding on IL-19 promoter to activate IL-19 expression due to DNA demethylation of IL-19 promoter

Combining UCSC (https://genome.ucsc.edu/) and JASPAR (http://jaspar.genereg.net/) public databases[33, 34], we hypothesized that the transcription factor Atoh1 could affect IL-19 expression. Firstly, we determined the mRNA and protein expression levels of Atoh1 in BMMs from young and old mice, but there were no differences in mRNA and protein levels (Fig. 5A-B). To further elucidate the role of Atoh1, we constructed Atoh1 overexpression plasmid vectors with HA tags, and transfected them into mouse BMMs. Western blot analysis confirmed the successful expression of exogenous HA-Atoh1 in mouse BMMs (Fig. 5C). At 48 hours post-transfection, we observed that overexpression of exogenous HA-Atoh1 protein in mouse BMMs increased the mRNA expression level (Fig. 5D) and secretion level (Fig. 5E) of IL-19. In addition, we utilized siRNA targeting Atoh1 to transfect into mouse BMMs to down-regulate Atoh1 expression and detected successful down-regulation of Atoh1 expression by western blot (Fig. 5F). In contrast to the results at overexpression, 48 hours post-transfection led to a decrease in both mRNA expression (Fig. 5G) and secretion levels (Fig. 5H) of IL-19 in mouse BMMs. These results together revealed that the modulation of Atoh1 levels directly correlates with changes in IL-19 expression and secretion, confirming a consistent trend.

To elucidate the underlying mechanism of the effect of Atoh1 on IL-19 expression, we assessed the binding level of Atoh1 to the promoter of the IL-19 gene in BMMs from both young and old mice. Notably, the binding of Atoh1 to the IL-19 promoter was substantially higher in BMMs from old mice compared with young mice (Fig. 5I). Furthermore, we examined the binding level of Atoh1 to the IL-19 gene promoter after 5’-aza treatment and overexpression of exogenous His-Dnmt1 of mouse BMMs. The results showed that the binding level of Atoh1 to the IL-19 gene promoter was significantly up-regulated after 5’-aza treatment (Fig. 5J), while the level was down-regulated after overexpression of exogenous His-Dnmt1 (Fig. 5K). In summary, our findings demonstrated that Atoh-1 bound to the IL-19 promoter to activacte IL-19 expression due to DNA demethylation of the IL-19 promoter.

**Figure 6.**
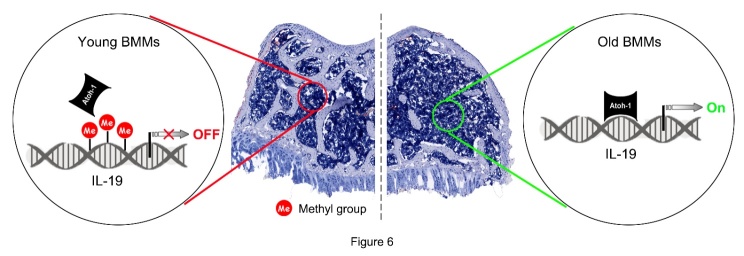
**Diagram for the mechanisms underlying DNA demethylation-mediated IL-19 expression activation in BMMs from old mice**. In young BMMs, IL-19 expression is maintained in the low level, because DNA hyper-methylation in IL-19 promoter prevents Atoh-1 from binding on IL-19 promoter. In old BMMs, DNA in IL-19 promoter is de-methylated, which activates IL-19 expression via facilitating Atoh-1 binding on IL-19 promoter. As a result, osteoclastic differentiation potential is elevated in old BMMs and bone loss is generated in old skeleton.

### DISCUSSION

The current investigation reveals that in old mice, both the expression and secretion of IL-19 are augmented, contributing to the observed bone loss within this demographic. This increase is associated with hypomethylation levels in the promoter region of the IL-19 gene, with Dnmt1 playing a predominant role in this process. This process is associated with hypomethylation levels in the promoter region of the IL-19 gene in old mice, in which Dnmt1 plays a predominant role. Through further studies, we find that hypomethylation levels of the IL-19 promoter region in BMMs of old mice promote binding of Atoh1 to the IL-19 promoter and activate IL-19 expression. Nevertheless, in BMMs from young mice, IL-19 expression is maintained in the low level, because DNA hyper-methylation in IL-19 promoter prevents Atoh-1 from binding on IL-19 promoter (Fig. 6).

IL-19, a cytokine exhibiting both pro-inflammatory and anti-inflammatory properties, plays a complex role in age-related bone loss, potentially involving a variety of inflammatory mediators and multiple biological pathways. In intervertebral disc cells, IL-19 has been implicated in the pathogenesis of degenerative lumbar spondylolisthesis through the induction of pro-inflammatory cytokine expression [35]. For osteoclast differentiation, IL-19 has been shown to stimulate the secretion of TNF-α, IL-1β, and IL-6, as well as augment the expression of RANKL in synovial fibroblasts, thereby enhancing osteoclastogenesis [36]. Conversely, *in vitro* studies have suggested that IL-19 may inhibit osteoclast differentiation by attenuating NF-κB and p38 MAPK activation, along with reducing c-Fos expression in the mouse macrophage-like cell line RAW264.7 [37]. We hypothesize that the effects of IL-19 on bone metabolism is multifaceted and indirect, likely engaging various cell types and multiple mechanisms. Consequently, we have opted for *in vivo* animal experiments to more accurately ascertain the influence of IL-19 on bone metabolism. Our study shows the high expression and secretion levels of IL-19 in old mice, and in vivo experiments determine that high IL-19 levels have a negative impact on bone mass and bone microarchitecture as well as increase the number of osteoclasts.

Given that DNA methylation is involved in establishing patterns of gene repression during development, we investigated whether DNA methylation levels play a role in IL-19 expression levels in old mice [38]. Our results confirm that high IL-19 levels in old mice are associated with low methylation levels of the IL-19 promoter region in BMMs. Among the Dnmts responsible for catalyzing DNA methylation, Dnmt3a and Dnmt3b are the main enzymes that establish DNA methylation during embryonic developmecnt. Dnmt3l, despite lacking catalytic activity, plays a crucial auxiliary role in de novo methylation. Once established, the methylation pattern is maintained during DNA replication by the DNA methyltransferase Dnmt1 [31]. Our findings reveal that in aged mice, there is a notable reduction in both the mRNA expression of Dnmt1 and its binding affinity to the IL-19 gene promoter, while the levels of Dnmt3a, 3b, and 3l remain stable. These results corroborate the influence of Dnmt1 expression on IL-19 levels. In other words, the high IL-19 levels in old mice are due to the low expression of Dnmt1 during DNA replication that prevents methylation levels from being fully maintained.

Atoh1 is a basic helix-loop-helix transcription factor with multiple targets but high specificity [39-41]. Gene ontology (GO) annotations associated with Atoh1 include DNA-binding transcription factor activity and sequence-specific DNA binding at the cis-regulatory region of RNA polymerase II (https://www.ncbi.nlm.nih.gov/). It has been shown that DNA methylation overlaped with the binding site of Atoh1 and that DNA hypermethylation at regulatory elements can achieve gene silencing by preventing ATOH1 binding [42]. Additionally, it has also been suggested that Atoh1 may interact with N-methyltransferases[43]. Numerous studies have demonstrated that Atoh1 plays an important role in the development of the nerve, gut and inner ear, but its involvement in bone metabolism remains unexplored [43-45]. Here, we predict and demonstrate that methylation levels in the mouse IL-19 promoter region regulate IL-19 expression levels by influencing the binding of Atoh1 to transcription sites in the IL-19 promoter region, which in turn acts on the regulatory mechanism of bone metabolism. As a result, osteoclast differentiation potential is elevated in old mice, which in turn produces bone loss.

There are some limitations that need to be mentioned here. Firstly, the effects of IL-19 on bone remain complex and depend on many factors such as different receptors, multiple cells, and interactions with other cytokines. Since aging is an important factor in promoting the activation of inflammatory responses and is strongly associated with bone loss, the role of IL-19 in aging-associated bone metabolism is likely to be related to inflammation [46, 47]. Consequently, additional research is imperative to ascertain whether the involvement of IL-19 in age-related osteoporosis is connected to inflammatory mechanisms. In addition, this study focused on BMMs and osteoclasts to affect bone metabolism, and its downstream mechanism of action still needs to be further explored. Finally, the findings and mechanisms elucidated in this study are limited to animal and cell experiments, and their applicability in humans needs to be further studied and confirmed.

Our study confirms that high expression of IL-19 promotes aging-induced bone loss in aged mice and elucidates that the upstream mechanism regulating IL-19 expression and secretion is mainly the methylation level of the promoter region of the IL-19 gene that affects the binding of Atoh1 to the promoter region. The results of the present study make targeting IL-19 a direction for regulating bone loss-related diseases and help to improve the current understanding of the mechanisms by which DNA methylation levels influence aging-associated bone loss. Future studies can focus on the comprehensive mechanism of IL-19 action on bone metabolism and bone microenvironment and verify the expression and role of IL-19 in human body. More importantly, it is expected to increase the DNA methylation level of IL-19 promoter region through drug delivery, and then reduce the secretion level of IL-19, which has good clinical translational significance for senile bone loss.

### Conclusion

The IL-19 promoter region in the DNA of old mice's BMMs is demethylated, leading to activation of IL-19 expression by enhancing the binding of Atoh-1 to the IL-19 promoter. High expression of IL-19 promotes osteoclast differentiation, which in turn induces bone loss in old mice, and participates in the onset and progression of age-related bone loss. Consequently, IL-19 emerges as a promising therapeutic target for the management of osteoporosis.
